# Lack of furosemide responsiveness predict severe acute kidney injury after liver transplantation

**DOI:** 10.1038/s41598-023-31757-8

**Published:** 2023-03-27

**Authors:** Li Cai, Liu Shu, Zhao Yujun, Cheng Ke, Wang Qiang

**Affiliations:** 1 Department of Transplantation, The Third Xiangya Hospital, Central South University, Changsha, China; 2 Engineering and Technology Research Center for Transplantation Medicine of National Health Comission, The Third Xiangya Hospital, Central South University, Changsha, China

**Keywords:** Biomarkers, Gastrointestinal diseases, Kidney diseases

## Abstract

Acute kidney injury (AKI) remains to be a common but severe complication after liver transplantation (LT). However, there are still few clinically validated biomarkers. A total of 214 patients who underwent routine furosemide (1–2 mg/kg) after LT were retrospectively included. The urine output during the first 6 h was recorded to evaluate the predictive value of AKI stage 3 and renal replacement therapy (RRT). 105 (49.07%) patients developed AKI, including 21 (9.81%) progression to AKI stage 3 and 10 (4.67%) requiring RRT. The urine output decreased with the increasing severity of AKI. The urine output of AKI stage 3 did not significantly increase after the use of furosemide. The area under the receiver operator characteristic (ROC) curves for the total urine output in the first hour to predict progression to AKI stage 3 was 0.94 (*p* < 0.001). The ideal cutoff for predicting AKI progression during the first hour was a urine volume of less than 200 ml with a sensitivity of 90.48% and specificity of 86.53%. The area under the ROC curves for the total urine output in the six hours to predict progression to RRT was 0.944 (*p* < 0.001). The ideal cutoff was a urine volume of less than 500 ml with a sensitivity of 90% and specificity of 90.91%. Severe AKI after liver transplantation seriously affects the outcome of patients. Lack of furosemide responsiveness quickly and accurately predict AKI stage 3, and patients requiring RRT after the operation.

## Introduction

Liver transplantation (LT) is the best option for the treatment of end-stage liver disease, its efficacy, and long-term survival have improved significantly in the last two decades. However, acute kidney injury (AKI) remains to be a common but serious complication after liver transplantation^1^, and the prevalence is as high as 40.7%. The incidence of severe AKI requiring renal replacement therapy (RRT) is up to 7.7%^2^. Numerous studies have shown that AKI affects graft survival and patient prognosis, and also increases the length of hospital stay, resource utilization, and medical expenses^3^.

The mechanism of AKI after LT is currently unclear. It may be related to reperfusion syndrome^3^, internal kidney damage caused by preoperative liver dysfunction, hormone imbalance, infection, use of nephrotoxic drugs, surgery, renal ischemia–reperfusion injury, and inflammation^4–6^. Due to the lack of effective treatments, timely identification and detection of early AKI stage is crucial. The discovery of biomarkers may not only enable early diagnosis and reverse AKI, but also assess the duration and prognosis of renal impairment^7^. There are many serum biomarkers available for the diagnosis and prognosis determination of AKI, and these markers may also serve as therapeutic targets for alleviating kidney injury, blocking liver ischemia–reperfusion injury, and improving postoperative hepatocytes regeneration^8–10^. Because the pathogenesis of AKI after LT is currently not available, and different fluid loads, and the measurement of biomarkers at different time points can also impact outcomes, there is still few clinically validated biomarkers^11,12^.

Furosemide is the most commonly used loop diuretic in the clinical practice, which mainly inhibits the active reabsorption of NaCl in the medullary loop thick wall segments of renal tubules, increasing the intraluminal Na^+^ and Cl^−^ concentrations, while decreasing the medullary interstitial Na^+^ and Cl^−^ concentrations^13^. It can reduce the osmotic pressure gradient and decrease the tubular concentrating function, thus leading to increased water, Na^+^, and Cl^−^ excretion^14^. Based on the pharmacological properties of furosemide, the induced urine volume can be used to predict renal tubular integrity and as a marker to evaluate the severity of AKI^15^. This method was first proposed by Baek et al. in 1973, and was standardized by Chawla et al. in 2013 as the furosemide stress test (FST)^16^. Previous studies have shown that the FST better predicted the onset of AKI stage 3 in patients and had better actionability compared to other common serum biomarkers^17^. The FST can also be used to screen high-risk patients who require blood purification therapy and to determine the timing to initiate RRT, and it can even be used to predict the recovery of renal function in patients with severe AKI and the timing to terminate RRT^18–20^. Because the pathogenesis of post-LT AKI stage is different from that of other diseases, it is unclear whether furosemide use has the same efficacy and safety profile for post-LT AKI.

The main objective of this retrospective study was to evaluate the role of furosemide in the evaluation of post-LT AKI. In addition, we evaluated the predictive role for RRT.

## Materials and methods

### Research subjects

**Figure 1:**
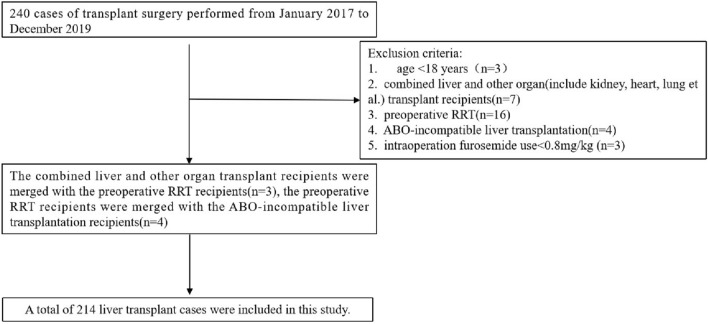
Patients flow.

Patients who underwent allogeneic liver transplantation at the Third Xiangya Hospital of Central South University from January 2017 to December 2019 were included in this study (n = 240), and exclusion criteria included: 1. Age < 18 years (n = 3); 2. Combined liver and other organ (include kidney, heart, lung et al.) transplant recipients (n = 7); 3. Preoperative RRT (n = 16); 4. ABO-incompatible liver transplantation (n = 4); 5. Intraoperation furosemide use < 0.8 mg/kg (n = 3). There is an overlap between the exclusion criteria 2 and 3, 3 and 4. A total of 214 liver transplant cases were included in this study (Fig. 1).

### Ethics statement

The manuscript complies with current regulations and the Declaration of Helsinki, and has been approved by the IRB of the Third Xiangya Hospital of Central South University (the ethics approval number is No 21144). The informed consent was obtained.

### Perioperative management

Grafts were derived from circulating postmortem donors (DCD), or brain postmortem donors (DBD). No organs from executed prisoners were used. All patients received a modified piggyback liver transplantation, a technique of side-to-side caval anastomosis during orthotopic hepatic transplantation without inferior vena caval occlusion. The portal-systemic venous shunt technique was not used. The use of furosemide was a routine process after inferior vena cava opening immediately according to the weight, mostly 60–120 mg. The precision urine output per hour was accurately recorded by nurses. Induction therapy with basiliximab was used intraoperatively, and calcineurin inhibitors (CNIs) were used within 12 h after the operation in the patients without preporative renal dysfunction. For patients with preoperative renal insufficiency, the use of CNIs starts from a small dose and gradually increases to maintain the tacrolimus valley concentration to 4–6 ng/ml. RRT was initiated only if one of the following criteria were met: blood urea nitrogen ≥ 100 mg/dL, serum potassium > 6.5 mmol/L, serum bicarbonate < 12 mmol/L or pH < 7.15, or pulmonary edema. Kidney function test was performed everyday within 7 days after the operation.

### Definition of AKI

The primary outcome was the occurrence of postoperative AKI, which was defined as positive when serum creatinine increased by 0.3 mg/dL or more within postoperative day (POD) 2 or by a factor of 1.5 or more within POD 7, according to the creatinine criteria of the Kidney Disease: Improving Global Outcomes (KDIGO) classification^21^. It is further divided acute kidney injury into the following three grades: grade 1 is defined as an increase in creatinine ≥ 0.3 mg/dL within POD 2 or 1.5 ~ 1.9 fold increase over baseline creatinine within POD 7 or urine output < 0.5 ml/(kg*h) last 6 ~ 12 h; grade 2 is defined as an increase in creatinine 2.0 ~ 2.9 fold from baseline creatinine within POD 7 or urine output < 0.5 ml/(kg*h) last more than 12 h; and grade 3 is defined as when an increase in creatinine is more than threefold over baseline creatinine within POD 7, a creatinine value ≥ 4.0 mg/dL, and an acute increase of at least 0.5 mg/dL within POD 7, or patients receiving renal replacement therapy within POD 7, or urine output < 0.3 ml/(kg*h) last more than 12 h, or anuria > 12 h.

### Data collection and statistics

Patients’ general data and clinical characteristics were extracted and recorded from the EMRs system. Preoperative statistics included gender, age, height, weight, cause of disease, hypertension, comorbidities, total bilirubin, creatinine, international normalized ratio (INR), eGFR (MDRD), hemogram. Intraoperative statistics include operative time (from skin incision to end of skin suture), intrahepatic inferior vena cava block time, estimated blood loss, and the amount of RBC transfusion. Donor-related data were collected from the cortis system, including gender, age, height, weight, primary disease, alanine aminotransferase (ALT), aspartate aminotransferase (AST), and albumin. Assessment of the degree of the donor liver steatosis was obtained from the pathology department, and all the donor livers were subjected to rapidly frozen biopsy before operation but reconfirmed by postoperative paraffin sections.

Continuous variables were described by mean ± SD or median (interquartile range), and percentages were calculated for categorical variables. Analyses between groups were performed using the Student’s *t*-test, analysis of variance for continuous variables, and χ^2^ test or Fisher’s exact test for categorical variables. Postoperative survival was estimated by Kaplan–Meier method with the Gehan-Breslow-Wilcoxon test. ROC curve was used for the prediction of AKI, *Delong’s* test was used to compare every two ROCs. Finally, all *p* values were 2-sided, and a *p* value of < 0.05 was considered statistically significant, with an alpha value of 0.05 and a beta value of 0.1. SPSS version 22.0 (IBM Corporation, Armonk, NY) and Medcalc (MedCalc Software Ltd) were used for analysis.

## Results

### Basic characteristics

In total, 214 liver transplant recipients were included in this study, of whom a total of 105 (49.07%) developed AKI. Recipients who developed AKI had a lower mean age (44.83 ± 10.88 vs. 48.29 ± 10.13, *p* < 0.05) and BMI (22.86 ± 2.94 vs. 23.95 ± 3.31, *p* < 0.05) than recipients who did not develop AKI. Recipients in the AKI group had higher preoperative serum creatinine value levels than those in the control group (86.03 ± 43.81 vs. 71.97 ± 23.16, *p* < 0.05). Non AKI group had higher eGFR than AKI group (110.63 ± 38.23 vs. 97.89 ± 41.35, *p* < 0.05). Meanwhile, the intraoperative blood loss and intraoperative RBC transfusion were higher in recipients with AKI than in the control group (2335.71 ± 1946.55 vs. 1767.89 ± 1359.93, *p* < 0.05; 2445.05 ± 1816.11 vs. 1751.63 ± 1369.47, *p* < 0.05). Donor-related factors were also associated with the occurrence of AKI, with the mean values of AST being higher in donors in the AKI group than in controls (110.63 ± 191.19 vs. 54.32 ± 41.22, *p* < 0.05) (Table 1).

**Table 1:** Basic characteristics of the perioperative period of the recipient.

	Non AKI (n = 109)	AKI (n = 105)	*p*
Pre-operation			
Sex			
Male	93 (85.3)	93 (88.6)	0.481
Age (years)	44.83 ± 10.88	48.29 ± 10.13	0.017
BMI (kg/m^2^)	22.86 ± 2.94	23.95 ± 3.31	0.011
Etiology			
Virus hepatitis	86 (88.9)	85 (80.9)	0.628
Alcoholic hepatitis	6 (5.5)	6 (5.7)	
Autoimmune liver disease	7 (6.4)	3 (2.9)	
Drug-induced liver failure	1 (0.9)	3 (2.9)	
Others	9 (8.3)	8 (7.6)	
MELD	19 (3–34)	20 (5–36)	0.190
Diabetes	17	27	0.067
Hypertension	18	15	0.652
Hemogram			
WBC (10^9^/L)	6.72 ± 3.76	5.82 ± 3.27	0.065
Hb (g/L)	110.78 ± 25.23	106.30 ± 22.43	0.172
Plt (10^9^/L)	93.60 ± 77.00	76.87 ± 53.30	0.067
TBIL (μmol/L)	271.46 ± 237.80	266.80 ± 239.49	0.887
Serum creatinine (μmol/L)	71.97 ± 23.16	86.03 ± 43.81	0.002
eGFR (MDRD) ml/min/1.73m^2^	110.63 ± 38.23	97.89 ± 41.35	0.02
INR	1.98 ± 0.841	2.12 ± 1.14	0.252
Albumin (g/L)	34.79 ± 5.77	34.97 ± 11.13	0.885
Intra-operation			
Anhepatic phase (min)	67.65 ± 17.91	70.90 ± 14.70	0.475
Operation time (hours)	6.22 ± 1.02	6.23 ± 1.00	0.978
Blood loss (ml)	1767.89 ± 1359.93	2335.71 ± 1946.55	0.017
Red cell transfusion (ml)	1751.63 ± 1369.47	2445.05 ± 1816.11	0.010
Donor factors			
Age (Years)	41.94 ± 11.86	41.02 ± 12.46	0.582
BMI (kg/m^2^)	21.78 ± 2.92	22.10 ± 3.03	0.422
ALT (U/L)	59.09 ± 80.67	99.87 ± 165.20	0.076
AST (U/L)	54.32 ± 41.22	110.63 ± 191.19	0.023
Albumin (g/L)	35.65 ± 7.43	35.71 ± 7.59	0.963
CIT (hours)	8.15 ± 2.01	8.15 ± 2.20	0.998
Primary disease			
Brain trauma	52	55	0.263
Cerebral apoplexy	49	40	
Brain tumor	8	7	
Others	0	3	
Degree of steatosis			
Light	74	73	0.910
Mild	19	16	
Severe	16	16	
Stage of AKI			
Stage 1	/	59 (56.2%)	/
Stage 2	/	25 (23.8%)	
Stage 3	/	21 (20.0%)	
RRT		10 (9.5%)	/
Furosemid dosage (mg/kg)	1.51 ± 0.34	1.45 ± 0.29	0.170

*BMI* body mass index, *MELD* model for end-stage liver disease score; *AKI* acute kidney injury, *eGFR* estimated glomerular filtration rate, *WBC* white blood cell, *Hb* hemoglobin, *Plt* pletelet, *TBIL* total bilirubin, *INR* international normalized ratio, *ALT* alanine aminotrans, *AST* aspartate aminotransferase, *CIT* cold ischemia time, *AKI* acute kidney injury, *RRT* renal replacement therapy.

### Comparison of different indexes and prognosis between non AKI and different AKI stages

**Figure 2:**
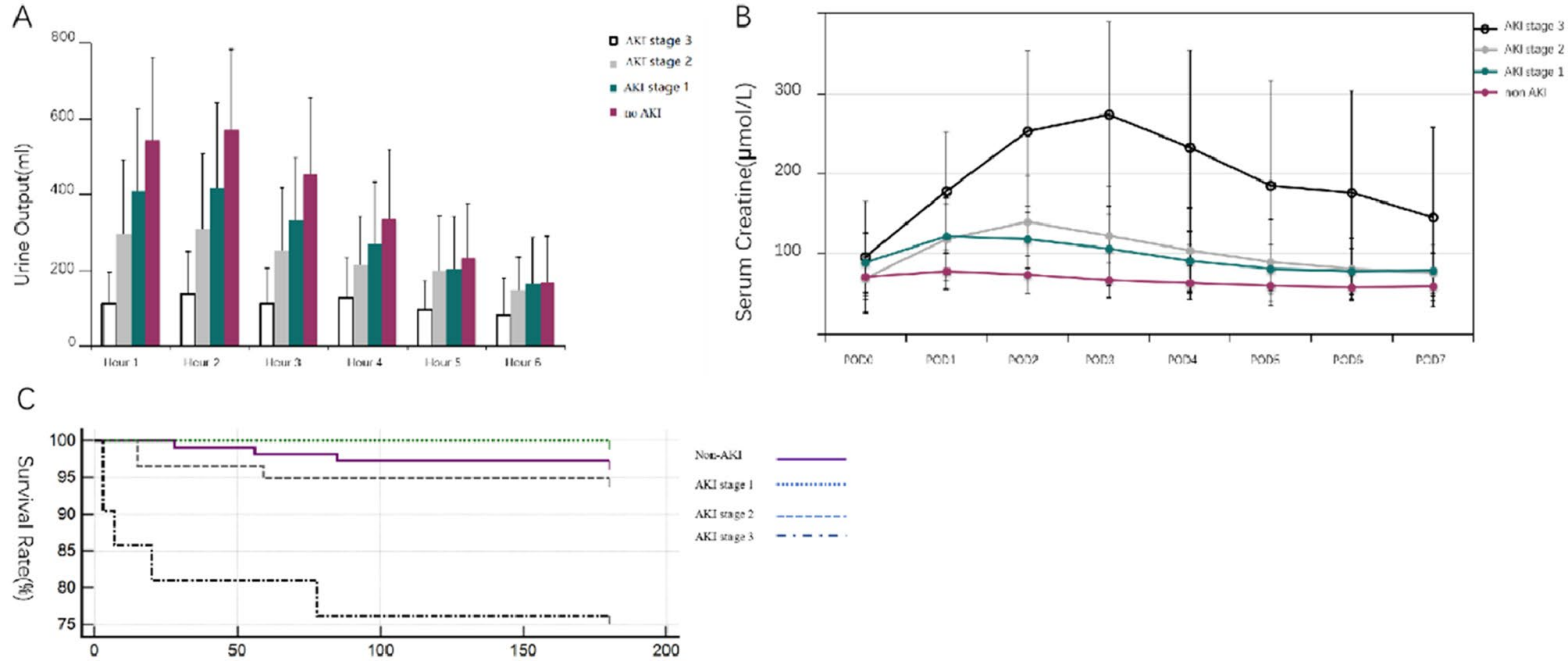
(**A**) Urine output during the first 6 h after the FST. The urine output per hour was recorded accurately; (**B**) Creatinine before and within 7 days after liver transplantation (*POD* post-operation day; (**C**) Survival curves of recipients at different AKI stages. The survival outcome after the first six mouths was significantly in AKI stage 3 than in the other group (*p* < 0.05).

To clarify the impacts of different degrees of AKI on liver transplant recipients, all recipients were divided into 4 groups according to KDIGO criteria: non AKI, AKI stage 1, AKI stage 2, AKI stage 3. Comparing the urine output among the 4 groups revealed that the urine output decreased with the increasing severity of AKI within 3 h after the use of furosemide, all with statistically significant differences (*p* < 0.05). Urine output in the non AKI and AKI stage 1 peaked at 2 h and then decreased rapidly because of the weakened utility of furosemide. The urine output of AKI stage 3 did not significantly increase, and all remained low at 6 h (Fig. 2A, Tables S1 and S2). There was no significant difference in urine volume among the first 3 groups at 5 and 6 h (*p* > 0.05), however, the urine volume of patients with AKI stage 3 was significantly lower than that of the first 3 groups (*p* < 0.05) (Table S2). Renal function was monitored daily postoperatively for all recipients, and creatinine increased significantly for those in AKI stage 3, reaching its highest value on POD 3, and then decreased slowly. For AKI stage 1 and 2 recipients, high creatinine values were reached on postoperative days 1 and 2, followed by a rapid decrease to preoperative levels. For recipients in the non AKI group, the creatinine increased slightly or did not increase compared to all the AKI recipients. At the same time, they maintained a more stable level after surgery. The daily creatinine of AKI stage 3 recipients were higher than those of the other 3 groups (*p* < 0.001) (Fig. 2B and Table S3).

A total of 11 recipients died within 6 months after operation, of which 2 died from gastrointestinal hemorrhage, 3 from cerebrovascular accident, 1 from GVHD, and 5 from infection (pulmonary infection or sepsis). The 6-month postoperative survival rates of the 4 groups were compared and a survival curve was plotted (Fig. 2C). The postoperative 6-month survival rate in non AKI group (97.2%) did not differ significantly from the AKI stage 1 and 2 (100% and 96.4%, respectively) (*p* > 0.05), however, it was lower in the AKI stage 3 than in the first 3 groups (*p* < 0.05). It showed that severe AKI has a great impact on patients’ outcomes, and early identification of severe AKI may be extremely valuable in improving their survival.

### FST has a good predictive effect in patients at AKI stage 3

**Figure 3:**
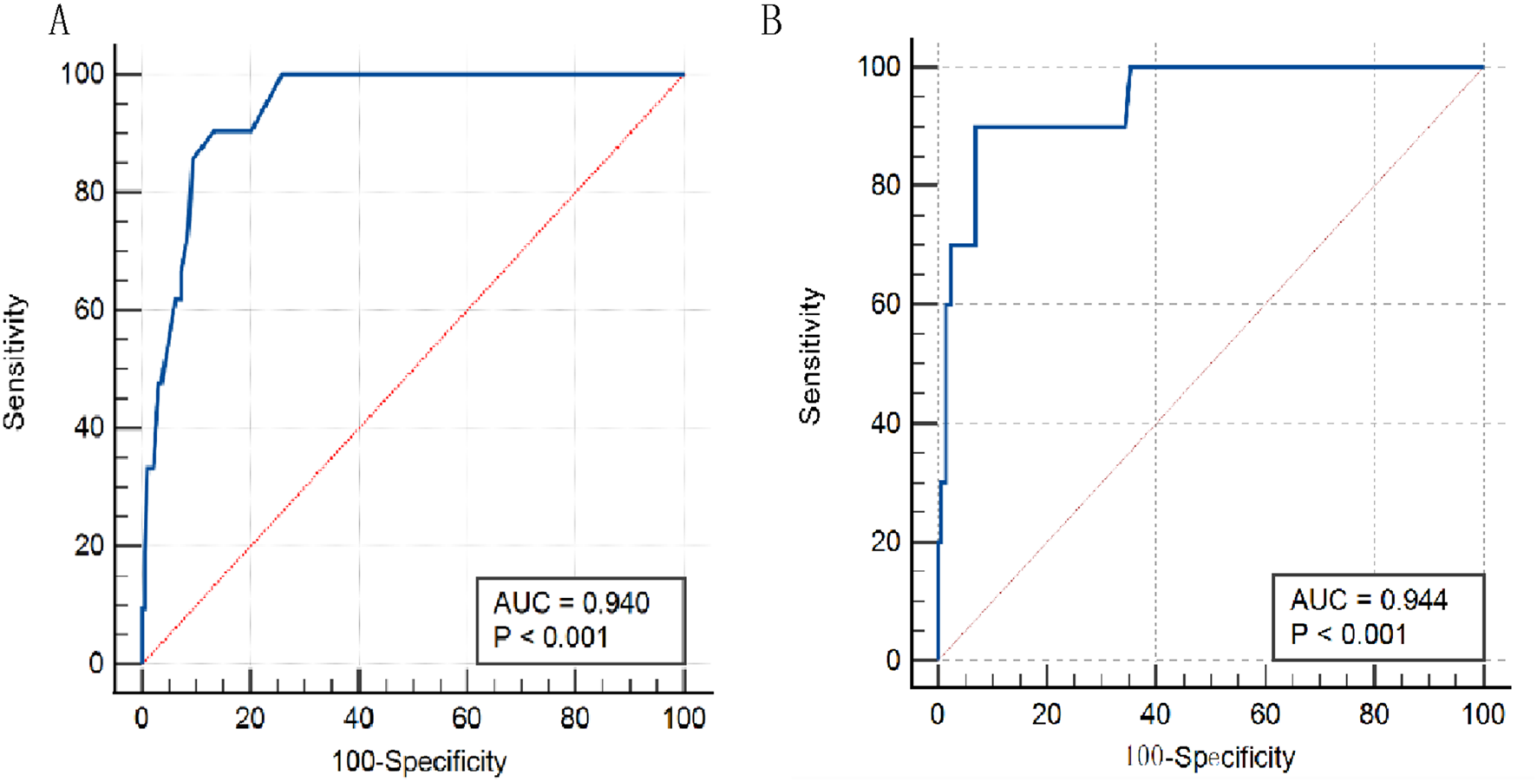
(**A**) Urine output in the first hour for prediction of progression to AKI stage 3 after LT. The area under the ROC curve was 0.940 (95% CI 0.905–0.976, *p* < 0.001); 3(**B**)Total six-hour urine output for prediction of progression to RRT after LT. The area under the ROC curve was 0.944 (95% CI 0.88–1.00, *p* < 0.001).

We calculated the total urine volume from the beginning to different nodes after FST, using the hour as the node. Employing these variables to draw ROC curves for predicting AKI stage 3, we found that the area under the curve exceeded 0.90 for nearly every variable (Table 2). *Delong’s* test for every two variables showed no significant difference (*p* > 0.05) between all variables (Table S6), indicating that FST is an excellent predictor on AKI stage 3. The area under the curve at 1 h was the best (AUC = 0.94), and further we found that urine output in 1 h < 200 ml had the best predictive value with a sensitivity of 90.48% and specificity of 86.53% (Table 2 and Fig. 3A). Similarly, we also used the same variables to draw the ROC curve for the whole AKI, and urine output within 2 h had the best predictive value (AUC = 0.782) (Tables S4 and S5).

**Table 2:** Predictive value of different variables on AKI stage 3.

AUCs for prediction of progression to AKI stage 3				
	AUC	*p*	95% CI	
			Lower	Upper
1 h	0.940	< 0.001	0.905	0.976
2 h	0.935	< 0.001	0.893	0.976
3 h	0.935	< 0.001	0.893	0.976
4 h	0.927	< 0.001	0.882	0.972
5 h	0.926	< 0.001	0.882	0.969
6 h	0.919	< 0.001	0.868	0.97

Sensitivity and specificity of 1 h urine output for progression to AKI stage 3				
	Sensitivity	Specificity	+ LR	− LR
≤ 50	33.33	98.96	32.17	0.67
≤ 100	61.9	93.78	9.96	0.41
≤ 150	76.19	91.19	8.65	0.26
≤ 200	90.48	86.53	6.72	0.11
≤ 250	90.48	79.79	4.48	0.12
≤ 300	100	74.09	3.86	0

### FST has a good predictive value for RRT

We evaluated the predictive role of FST on RRT after LT. 10 of 21 patients with AKI stage 3 were treated with RRT, and plotting ROC curves suggested a good predictive value (AUC > 0.90) for all variables, with the best predictive value (AUC = 0.944) for 6 h urine output (Table 3), and 6 h urine output < 500 ml had the best Youden index (sensitivity = 90%, specificity 90.91%) (Table 3 and Fig. 3B).

**Table 3:** Predictive value of different variables on RRT.

AUCs for prediction of progression to RRT				
	AUC	*p*	95% CI	
			Lower	Upper
1 h	0.909	< 0.001	0.852	0.966
2 h	0.921	< 0.001	0.863	0.978
3 h	0.927	< 0.001	0.871	0.983
4 h	0.934	< 0.001	0.863	1
5 h	0.942	< 0.001	0.877	1
6 h	0.944	< 0.001	0.88	1

Sensitivity and specificity of six-hour urine thresholds for progression to AKI stage 3				
	Sensitivity	Specificity	+ LR	− LR
≤ 200	20	100		0.8
≤ 300	20	99.51	40.8	0.8
≤ 400	60	98.53	40.8	0.41
≤ 500	90	93.14	13.11	0.11

## Discussion

In this retrospective study, we aimed to evaluate the predictive value of the use of furosemid for AKI after liver transplantation.

According to our results, AKI after liver transplantation critically affects patient outcomes. However, the 6-month postoperative survival between AKI stage 1 and 2 and non AKI was not significantly different, and only AKI stage 3 had a significant impact on the outcome of the recipients. Therefore, how quickly AKI stage 3 is diagnosed is extremely important for subsequent treatment. Both the widely used KDIGO guidelines and the Risk, Injury, Failure and Loss, and End-Stage(Rifile) criteria adopt creatinine and urine volume as indicators for renal function evaluation^22^. Creatinine has been used uniformly to classify renal function in most previous retrospective studies, however, these studies have a large heterogeneity, such as the time point of serum collection, the criteria of the assay, and inconsistent timing. Also, creatinine is influenced by diet, volume overload, body muscle mass, and liver function. Therefore, creatinine is used more as a marker of kidney function rather than a marker of kidney damage^23^. Creatinine is used to determine renal function in many cases, but it is not able to adapt timely to clinical needs. In recent years, many investigators worldwide have identified a variety of blood and urinary biomarkers for predicting AKI, such as NGAL, KIM-1, IGFBP7, L-FABP, netrin-1, sema-3a, ET-1, and so on^4,24^. There is currently no consensus on whether these biomarkers can predict the occurrence of AKI, and their detection is cumbersome, which is more accurate to predict AKI by the dynamic changes. So there is a need for a simple, rapid, and convenient method in clinical practice.

Furosemide is one of the most clinically used drugs, and its pharmacology, pharmacokinetics, and adverse effects have been well described in related disorders. More than 50 years ago, Baek et al*.* showed that urine output after furosemide administration could be used as a diagnostic criterion for acute tubular necrosis^15^. Ten years ago, Chawla devised a standard method to test the diagnostic role of furosemide in critical AKI, in which method, patients were administered a dose of 1 mg/kg, or 1.5 mg/kg in patients who had received furosemide within 7 days^16^. The primary diseases of major recipients in our study were acute on chronic liver failure, cirrhosis, or acute liver failure, almost all patients have used diuretics within 7 days before operation. Therefore, the dose of diuretic we used during the operation is higher than 1 mg/kg. The use of furosemide during the operation is to prevent renal tubular injury caused by long-term inferior vena cava occlusion, which has been the routine process in our center. After statistical analysis, we found that the use of large doses of furosemide can predict server AKI.

In the present study, we found that all variables after FST had important diagnostic value for AKI stage 3 (AUC > 0.9), and the urine volume in the first hour being the best predictor of AKI stage 3 (AUC = 0.94), significantly higher than other causes of AKI^16,17,25^. Also, the difference between our results and previous studies lies in the selected time point (1 h vs. 2 h). The difference in the results may be related to the difference in subjects studied. The inclusion criteria for the previous study subjects were patients admitted to ICU with AKI stage 1/2, while our subjects were all post-liver transplant patients, nearly half of whom had no AKI. These patients responded very quickly to furosemide, producing more urine in a short time. Theoretically, the duration of furosemide use in patients without AKI stage 3 is about 3 h. This phenomenon was also observed in our data. Starting from the 4th hour after FST, there was no significant difference in urine volume between the non AKI group and AKI stage 1 and AKI stage 2 (Table S2). However, the urine volume of these three groups remained significantly higher than that of AKI stage 3 at the 6th hour. These evidences show that the efficacy of furosemide decreases with the extension of time, and AKI stage 3 recipients basically have no response to furosemide, which may be due to the renal tubular damage. This suggests that FST provides us with evidence of unexplained baseline urinary flow rates, which may represent that FST evaluates the reserve function of the kidney.

RRT is the basic treatment for severe AKI after liver transplantation. However, clinicians disagree widely about the timing of initiating RRT. A large prospective double-blind controlled study showed that early initiation of RRT is beneficial in reducing 90-day mortality^26^. Early identification of high-risk patients and timely initiation of RRT treatment are of great significance to improve the survival rates of patients. In our study, only 10 patients need to receive RRT, much lower than other studies, which may be due to our center's strict indications for RRT^27,28^. Whether it is related to the use of furosemide needs to be further discussed. We found that urine volume < 500 ml within 6 h after FST had an excellent predictive value for RRT (AUC = 0.944, sensitivity 90%, specificity 90.91%), much higher than other biomarkers used in similar studies. However, we cannot determine whether early RRT is of positive significance in high-risk AKI patients after liver transplantation. Whether early RRT treatment has a positive effect on improving patient survival remains to be judged by further prospective studies.

It is also worth noting that, this is a single center retrospective study, which may be inconsistent with the treatment standards of other centers. For example, we have always been cautious about RRT, and the dose of furosemide during the operation was inconsistent (however, there is no significant difference in the dose of furosemide between the non AKI and AKI group). This study also has other limitations. Due to the lack of clinical data of some patients, there is no complete data of NGAL, KIM-1, IL-18, and other biomarkers to make a horizontal comparison with FST. Moreover, because of the limited number of patients with AKI stage 3, there is a lack of sufficient independent cohort to verify the effectiveness of the results. It needs to be verified by prospective clinical study.

## Conclusion

Severe AKI after liver transplantation seriously affects the outcome of patients. As a renal tubular function evaluation test, FST can quickly and accurately predict AKI stage 3, as well as RRT after the operation. At the same time, it is easy to operate. Further prospective studies are still needed to determine whether FST can be used as a starting standard of RRT after liver transplantation.

## Supplementary Information

Supplementary Information.

Supplementary Tables.

## Data Availability

The raw and analyzed datasets are included in the manuscript and supplementary files. However the raw datasets about liver donors are not publicly available due to data privacy laws, but are available from corresponding author on reasonable request.
