# Supplementary material for: Lack of furosemide responsiveness predict severe acute kidney injury after liver transplantation
Source: Sci Rep. 2023 Mar 27;13:4978. doi: 10.1038/s41598-023-31757-8 (PMC10042839; doi:10.1038/s41598-023-31757-8)
Supplement: Supplementary file 2 — Supplementary Tables. [file 41598_2023_31757_MOESM2_ESM.pdf]

Table S1 Urine volume after FST in different AKI stages and non-AKI group

	Hour 1(ml)	Hour 2(ml)	Hour 3(ml)	Hour 4(ml)	Hour 5(ml)	Hour 6(ml)
AKI 3	111.04± 84.49	138.33± 111.06	111.52± 93.60	127.33± 105.47	94.90± 75.51	82.38± 96.68
AKI 2	295.68± 196.83	309.6± 198.86	251.4± 166.76	214.4± 126.74	200.64± 143.62	149.08± 85.19
AKI 1	409.32± 217.74	418.72± 225.21	332.37± 164.86	270.67± 162.65	203.13± 139.57	162.03± 124.73
Non-AKI	544.31± 219.41	570.36± 212.84	452.70± 204.49	335.13± 183.99	232.70± 143.73	167.75± 122.57

Table S2 Difference of hourly urine volume after FST between different AKI stages

	1hUO	2hUO	3hUO	4hUO	5hUO	6hUO
AKI 3 VS AKI 2	<0.001	<0.001	<0.001	<0.05	0.004	0.02
AKI 3 VS AKI 1	<0.001	<0.001	<0.001	<0.001	0.001	0.019
AKI 3 VS non AKI	<0.001	<0.001	<0.001	<0.001	<0.001	0.003
AKI 2 VS AKI 1	<0.001	<0.001	<0.05	0.13	0.94	0.64
AKI 2 VS non AKI	<0.001	<0.001	<0.001	0.002	0.32	0.47
AKI 1 VS non AKI	<0.001	<0.001	<0.001	0.03	0.20	0.77

Table S3 Comparison of serum creatinine before operation and 7 days after operation in different AKI stages

	POD 0(μ mol/L)	POD 1(μ mol/L)	POD 2(μ mol/L)	POD 3(μ mol/L)	POD 4(μ mol/L)	POD 5(μ mol/L)	POD 6(μ mol/L)	POD 7(μ mol/L)
AKI 3	96.19 ± 68.93	178.42 ±74.05	252.80 ± 100.64	274.04 ± 114.77	232.5± 121.48	185.36 ± 130.24	176.36 ± 126.80	145.94 ± 112.04
AKI 2	69.76 ± 26.51	118.76 ±51.30	140.2± 58.07	122.36 ±61.60	104.68 ±52.71	89.56± 53.35	81.6± 37.50	76.36± 24.78
AKI 1	89.30 ± 36.74	121.72 ±40.34	118.18 ±42.42	106.47 ±43.42	91.40± 36.52	81.47± 30.55	78.64± 28.03	79.50± 31.62
Non -AKI	71.97 ± 23.16	78.88± 22.16	74.56± 23.03	67.44± 21.47	64.18± 20.77	60.43± 19.01	58.30± 15.74	59.69± 17.34

Table S4 ROC for prediction of AKI with urine output in different time point

	AUCs	*p* value	95%CI	
			Lower	Upper
1 hour	0.764	0	0.701	0.828
2 hours	0.782	0	0.721	0.843
3 hours	0.782	0	0.721	0.843
4 hours	0.780	0	0.719	0.841
5 hours	0.770	0	0.708	0.833
6 hours	0.758	0	0.694	0.821

Table S5 Difference of AUCs of variables for prediction of AKI

	1 hour	2 hours	3 hours	4 hours	5 hours	6 hours
1 hour	/	0.4824	0.5852	0.3264	0.3536	0.2869
2 hours	0.4824	/	1.0000	0.4822	0.5325	0.4133
3 hours	0.5852	1.0000	/	0.2339	0.3362	0.2368
4 hours	0.3264	0.4822	0.2339	/	0.8611	0.4290
5 hours	0.3536	0.5325	0.3362	0.8611	/	0.3237
6 hours	0.2869	0.4133	0.2368	0.2368	0.3237	/
